# Laparoscopic pancreaticoduodenectomy: are the best times coming?

**DOI:** 10.1186/s12957-019-1624-6

**Published:** 2019-05-10

**Authors:** Mengqi Liu, Shunrong Ji, Wenyan Xu, Wensheng Liu, Yi Qin, Qiangsheng Hu, Qiqing Sun, Zheng Zhang, Xianjun Yu, Xiaowu Xu

**Affiliations:** 1 Department of Pancreatic Surgery, Fudan University Shanghai Cancer Center, Shanghai, 200032 China; 2 Department of Oncology, Shanghai Medical College, Fudan University, Shanghai, 200032 China; 3 Pancreatic Cancer Institute, Fudan University, Shanghai Pancreatic Cancer Institute, Shanghai, 200032 China

**Keywords:** Laparoscopic, Pancreaticoduodenectomy, Open surgery, Robotic, Overall survival

## Abstract

**Background:**

The introduction of laparoscopic technology has greatly promoted the development of surgery, and the trend of minimally invasive surgery is becoming more and more obvious. However, there is no consensus as to whether laparoscopic pancreaticoduodenectomy (LPD) should be performed routinely.

**Main body:**

We summarized the development of laparoscopic pancreaticoduodenectomy (LPD) in recent years by comparing with open pancreaticoduodenectomy (OPD) and robotic pancreaticoduodenectomy (RPD) and evaluated its feasibility, perioperative, and long-term outcomes including operation time, length of hospital stay, estimated blood loss, and overall survival. Then, several relevant issues and challenges were discussed in depth.

**Conclusion:**

The perioperative and long-term outcomes of LPD are no worse and even better in length of hospital stay and estimated blood loss than OPD and RPD except for a few reports. Though with strict control of indications, standardized training, and learning, ensuring safety and reducing cost are still and will always the keys to the healthy development of LPD; the best times for it are coming.

## Background

The introduction of laparoscopic techniques in the 1980s heralded the start of revolutions in general surgical procedures and was performed in almost every abdominal surgery. Mounts of clinical trials were designed to provide evidence of laparoscopic safety and efficacy to inform clinical practices. Most studies comparing laparoscopy to traditional laparotomy have shown that laparoscopically resected patients experience less blood loss, postoperative pain and morbidity, shorter length of stay, improved cosmesis, and enhanced cost-effectiveness in multitude fields, including gastric, liver, and colon cancers[1–3]. Then, the last decade has seen the introduction of robotic assistance and more recently its application in many kinds of operations. Laparoscopic approaches to pancreatic surgery have not been widely adopted as the case in colorectal, urological, and gynecological surgeries, and almost all of laparoscopic pancreatic surgeries were performed in large, tertiary care centers. Though laparoscopy has been introduced in the field of pancreatic surgery for several decades, it mainly focused on the treatment of pancreatitis with pseudo-pancreatic cysts, laparoscopic exploration to assist pancreatic cancer staging, and advanced pancreatic tumors for palliative surgery and resection, etc.[4, 5]. While in the traditional sense of pancreatic surgery, such as operations like distal pancreatectomy, especially pancreaticoduodenectomy, it had been monopolized by open surgery, because of the multiple anastomoses reconstructions and large specimens during the operation, as well as because of concern for the potential for significant perioperative complications or death and the concern about adequate oncological outcomes when it is performed for malignant diseases [6].

However, after several decades of development, with the gradual deepening of the understanding of the anatomy of the pancreas, the emergence of a series of new surgical instruments, the application for laparoscopic pancreatic resection has been greatly broadened. Both laparoscopic distal pancreatectomy (LDP) and laparoscopic pancreaticoduodenectomy (LPD) are being performed more and more in the procedures of pancreatic surgery. And so far, LDP has already been widely adopted for benign and borderline tumors of the left pancreas, and meta-analysis shows that the minimally invasive advantage of LDP is obvious [7]. Many believe this approach can be the standard of care for benign and borderline tumors, even at an early stage of malignant tumors in the tail of the pancreas. However, there is no consensus as to whether LPD, which is a much more complex procedure, should be performed routinely. Herein, in this review, we summarized the development of LPD in recent years by comparing the mean operative time, estimated blood loss, length of hospital stay, and overall survival with open pancreaticoduodenectomy (OPD) and robotic pancreaticoduodenectomy (RPD) and evaluated its feasibility, perioperative, and long-term results.

## Main text

**Fig. 1:**
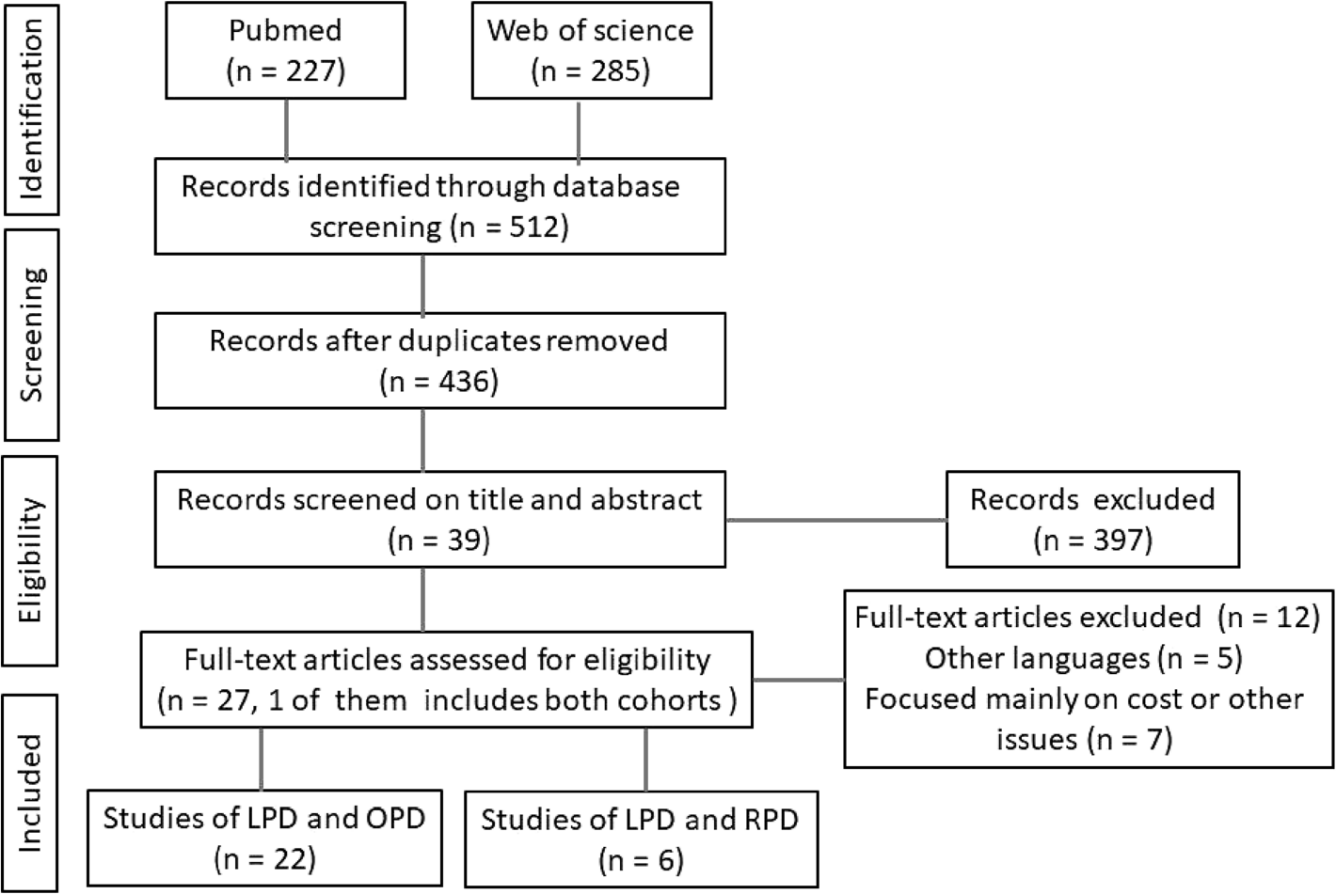
Flowchart of study screening according to PRISMA guidelines

The Preferred Reporting Items for Systematic Reviews and Meta-Analyses (PRISMA) were used in this review. A literature search was performed in the PubMed and Web of Science. The keywords were combined using Boolean operators as follows: ( Laparoscopic [Title] AND Pancreaticoduodenectomy [Title] ) AND ( Robotic [Title] AND Pancreaticoduodenectomy [Title] ). Inclusion criteria were studies comparing the perioperative and/or postoperative outcomes of LPD and OPD/RPD published between January 1, 2001, and December 31, 2018. Exclusion criteria were case reports, review articles, meta-analysis, studies comparing OPD and RPD only, and studies focusing on the cost or other non-medical issues (Fig. 1).

### Pancreaticoduodenectomy: laparoscopic versus open

Pancreaticoduodenectomy (PD), one of the subtlest and most complicated abdominal surgeries popularized by Whipple in 1935 [8], is the unique potential curative option for pancreatic cancer or periampullary malignancy. While for pancreatic cancer, apart from its deep location, even in the early stages, tends to metastasize and is more likely to invade its surrounding tissues or even major vessels around it. Therefore, PD was a surgery with high morbidity and mortality rate even in high-volume surgical institutions in the past. With the improvements in surgical techniques and perioperative care, significant advances have been achieved in PD over the past several decades and were widely accepted and performed on patients all around the world. In 1994, Gagner and Pomp first introduced laparoscopy to this most challenging operation [9]. Then, in 2007, Palanivelu et al. presented the first large series of LPD and proposed that not only was it possible but there may also be advantages in comparison to OPD in properly selected patients [10]. In 2011, a review of 27 published articles regarding LPD showed similar results with respect to perioperative morbidity and mortality rates compared to OPD [11]. Furthermore, several case series reported oncologic outcomes comparable to OPD in terms of consistent negative margin resection rates and lymph node retrieval [11–13]. A recent report analyzed 22,013 patients who underwent OPD or MIPD for pancreatic cancer using the National Cancer Database between 2010 and 2015, among which 3205 patients underwent LPD, and concluded that there was no difference in 90-day mortality between LPD and OPD, and the 30-day mortality, unplanned readmissions, margins, lymph nodes harvested, and receipt of adjuvant chemotherapy were equivalent between groups. Patients undergoing LPD were less likely to stay in the hospital for a prolonged time [14]. Meng et al. found that compared with patients who underwent OPD, patients who underwent LPD had a shorter time to first passage of flatus and oral intake, while no differences were seen in blood loss, length of intensive care unit stay, node positive, or R0 resection between the laparoscopic and open groups, and concluded that LPD may be a preferred method for surgeons to choose compared with OPD [15]. Lee and colleagues compared the perioperative outcomes of LPD and OPD after propensity score matching of 31 patients with benign and borderline malignant periampullary disease in each group and concluded that LPD shortened the length of hospitalization stay and minimized pain, with less blood loss during the operation in patients undergoing LPD than in those undergoing OPD, while no significant differences about major complications including the rate of postoperative pancreatic fistula [16]. More comparisons between LPD and OPD are listed in Table 1 [15–36].

**Table 1:** The comparisons between LPD and OPD from different aspects

Author	Study period (years)	Country	Study design	Number of patients LPD/OPD	Operation time, mean ± SD/median (range), (min) LPD/OPD	Length of stay, mean ± SD/median (range), (days) LPD/OPD	Estimated blood loss, mean ± SD/median (range), (ml) LPD/OPD	Pathology	Overall survival (month)/5-year survival rate
van Hilst et al. [24]	2016–2017	Dutch	PS,MC	50/49	410 (252–481)/274 (212–317)*	12 (7–21)/11 (7–24)	300 (200–438)/450 (300–1000)*	PDAC, IPMN, ampullary tumor, CC, DC, NET, other	–
Zhang et al. [17]	2014–2016	China	RS, SC	202/213	301 ± 175/320 ± 91	12.97 ± 7.21/19.18 ± 7.14*	194 ± 107/390 ± 301	–	–
Zimmerman et al. [28]	2014–2015	USA	RS, NSQIP	280/6336	421 (331.5–499)/354 (277–437.5)*	7 (6–12)/9 (7–13)*	–	–	–
Chen et al. [27]	2013–2017	China	RS, SC	47/55	410 ± 68/245 ± 70*	13 ± 4/18 ± 5.5*	210 ± 46/420 ± 50*	PDAC, IPMN, periampullary tumor, CC	–
Poves et al. [25]	2013–2017	Spain	PS, SC	32/29	486 (337–767)/365 (240–510)*	13.5 (5–54)/17 (6–150)*	–	PDAC, IPMN, ampullary tumor, SPT, NET, other	–
Palanivelu et al. [26]	2013–2015	India	PS, SC	32/32	359 ± 14/320 ± 13	7 (5–52)/13 (6–30)*	250 ± 22/401 ± 46*	Periampullary tumors	–
Meng et al. [15]	2010–2015	China	PS, SC	58/58	475.0 (420.0–546.3)/335.0 (275.0–405.0)*	14.0 (11.0–17.3)/13.0 (11.0–20.0)	200.0 (100.0–325.0)/220.0 (150.0–400.0)	Nonpancreatic periampullary adenocarcinomas	45/48
Tan et al. [18]	2009–2014	China	RS, SC	30/30	513.17 ± 56.13/371.67 ± 85.53*	9.97 ± 3.74/11.87 ± 4.72*	–	Mainly periampullary adenocarcinoma	–
Delitto et al. [19]	2010–2014	The USA	PS, SC	52/50	361 (–)/360 (–)	9.0 (–)/11.9 (–)*	260 (–)/518 (–)*	Periampullary adenocarcinoma	27.9/23.5
								PDAC	20.7/21.1
Sharpe et al. [33]	2010–2011	The USA	RS, NCDB	384/4037	–	10 ± 8.0/12 ± 9.7^*^	–	PDAC	–
Kantor et al. [31]	2010–2013	The USA	RS, NCDB	828/7385	–	10 ± 9/12 ± 9*	–	PDAC (stage I–II)	20.7/20.9
Chapman et al. [20]	2010–2013	The USA	RS, MC	248/1520	–	10 (7.0–15.0)/10 (7.0–15.5)	–	PDAC	19.8/15.6^#^
Langan et al. [35]	2010–2013	The USA	RS, SC	28/25	355/347	7.10/9.44*	336 (100–1400)/454 (100–1200)	PDAC, CC, other	–
Croome et al. [21]	2008–2013	The USA	RS, SC	108/214	379.4 ± 93.5/387.6 ± 91.8	6 (4–118)/9 (5–73)	492.4 ± 519.3/866.7 ± 733.7*	PDAC	25.3/21.8
Zureikat et al. [23]	2008–2010	The USA	RS, SC	14/14	456 (334–583)/372.5 (290–628)	8 (5–28)/8.5 (–)	300 (150–1300)/400 (–)	–	–
Song et al. [22]	2007–2012	South Korea	RS, SC	97/198	480.4 ± 116.4/351.9 ± 83.3*	14.1 ± 7.7/20 ± 10.7*	592 ± 376/555 ± 462	Benign or low-grade malignant tumors	–
				93/93	482.5 ± 117.6/347.9 ± 87.2*	14.3 ± 7.8/19.2 ± 8.8	609 ± 375/570 ± 448	PDAC and ampulla of Vater cancer	–
Asbun et al. [36]	2005–2011	The USA	RS, SC	53/215	541 ± 88/401 ± 108	8 ± 3.2/12.4 ± 8.5	195 ± 136/1032 ± 1151	PDAC, IPMN, other	–
Hakeem et al. [34]	2005–2009	The UK	RS, SC	12/12	–	14.9 ± 6.6/14.9 ± 5.7	–	Periampullary adenocarcinoma, common bile duct tumors	75%/50%
Khaled et al. [29]	2002–2015	The UK	PS, SC	15/15	470 (280-660)/310 (240-490)*	9.0 (7–20)/15.4 (8–49)*	300 (50-600)/620 (150–1200)*	Malignant lesions	67%/53%
Conrad et al. [30]	2000–2010	The USA	RS, SC	40/25	–	23 (9–311)/28 (11–179)	–	PDAC	35.5/29.6
Stauffer et al. [32]	1995–2014	The USA	RS, SC	58/193	518 (313–761)/375 (159–681)*	6 (4–68)/9 (4–71)*	250 (50–8500)/600 (50–7800)*	PDAC	32.07%/15.34%
Lee et al. [16]	1993–2017	South Korea	RS,SC	31/31	426.8 ± 98.58/355.03 ± 100.0*	14.74 ± 5.40/23.81 ± 11.63*	477.42 ± 374.80/800.00 ± 531.35*	Benign and borderline malignant Periampullary disease	–

–, no data

*PDAC* pancreatic ductal adenocarcinoma, *IPMN* intraductal papillary mucinous neoplasm, *SPT* solid papillary tumor, *CC* cholangiocarcinoma, *DC* duodenum carcinoma, *NET* neuroendocrine tumor, *PS* prospective study, *RS* retrospective study, *MC* multi-center, *SC* single center, *NSQIP* National Surgical Quality Improvement Program, *NCDB* National Cancer Database

*P < 0.05

^#^The study was performed among patients aged ≥ 75 years

### Pancreaticoduodenectomy: laparoscopic versus robotic

Since Himpens et al. introduced the first robotic surgery in cholecystectomy in 1997, the da Vinci robot had been applied in many surgical specialties such as gynecology and general surgery [37, 38]. However, until in 2010, the first manuscript report describing robotic pancreaticoduodenectomy was published by Giulianotti et al. [39], and then Nguyen and colleagues reported their experiences of the University of Pittsburgh [40]; both concluded that RPD/RAPD was safe and feasible if implemented reasonably. This opened the prelude to RPD; more and more centers carried out this procedure not only in benign disease processes and/or low-grade neoplasms but also in pancreatic cancer and periampullary malignancies and reported their data [41–44]. According to a recent report, Magge et al. found in a large series of RPD that the rate of postpancreatectomy hemorrhage (PPH), one of the most serious and life-threatening complications following PD, was similar to reported rates in historical open control series and pseudoaneurysm was less common with increasing experience [45]. In a subgroup analysis performed to compare outcomes following LPD versus RPD, the authors found there was no difference between LPD and RPD for short-term and oncologic outcomes, including operative time, estimated blood loss (EBL), length of postoperative hospital stay (LOS), and complication morbidities, but the rate of conversion from LPD to open is higher than RPD [14]. In another study comparing RPD and LPD using the pancreas-targeted American College of Surgeons National Surgical Quality Improvement Program (ACS-NSQIP), Nassour and colleagues [6] also concluded RPD was associated with a lower rate of conversion than LPD and a similar 30-day overall complication rate. However, there is also a study that reports that no obvious differences in the conversion rate between LPD (7%) and RAPD (8%) were seen [46]. Zhang et al. reviewed 20 RPD and 80 LPD performed in their institution and concluded that RPD and LPD had comparable short term results, but they found RPD seems to have a shorter learning curve than LPD for this complex procedure [47]. More comparisons between LPD and OPD are listed in Table 2 [6, 14, 28, 47–49].

**Table 2:** The comparisons between LPD and RPD from different aspects

Author	Study period (years)	Country	Study design	Number of patients LPD/RPD	Operation time, mean (median), min LPD/RPD	Hospital length of stay, mean (median) LPD/RPD	Estimated blood loss, mean (SD), ml LPD/RPD	Conversion rate	Overall survival (month)
Zhang et al. [47]	2013–2017	China	RS, SC	80/20	373.8 ± 70.2/407.0 ± 91.8	18.1 ± 11.6/14.6 ± 6.1	240 ± 239.5/220.5 ± 165.5	5%/0%	–
Liu et al. [48]	2015–2016	China	RS, SC	25/27	442 ± 96/387 ± 58*	24 ± 13/17 ± 5*	334 ± 175/219 ± 126*	4%/0%	–
Nassour et al. [6]	2014–2015	The USA	RS, NSQIP	235/193	429 (424)/422 (399)	10.6 (7.0)/10.7 (8.0)	-	26.0%/11.4%*	–
Zimmerman et al. [28]	2014–2015	The USA	RS, NSQIP	280/211	421 (331.5–499)/404 (331–511)	7 (6–12)/8 (6–11)	-	28%/11.4%*	–
Torphy et al. [14]	2010–2015	The USA	RS, MC	3205/549	–	–	-	25.5%/15.3%*	–
Nassour et al. [49]	2010–2013	The USA	RS, NCBD	1458/165	–	11.5/11.8	-	27.6%/17%*	20.7/22.7

–, no data.

*RS* retrospective study, *MC* multi-center, *SC* single center, *NSQIP* National Surgical Quality Improvement Program, *NCDB* National Cancer Database

*P < 0.05

While at present, most studies are accustomed to combining LPD and RPD as MIPD, and then compare MIPD with OPD, thus drawing conclusions that MIPD is superior to OPD in some aspects, or compare RPD/RAPD with OPD and draw conclusions [50–54]. Studies directly comparing robotic and laparoscopic approaches and concluding objective evidence of the advantages/disadvantages of the RPD over LPD are still lacking.

## Discussion

As more and more laparoscopic pancreatic surgeries being performed, encouraging data has emerged. With the help of new surgical equipment, laparoscopic surgeons become more skilled and gain more experiences, and patients are being benefited by this expanding technology. After decades of development, there is no doubt that the technique is now mature and the operation is safe and reliable. However, the main concern of doctors and patients remains whether LPD can achieve similar oncology outcomes as OPD, including progression-free survival and overall survival.

To date, in the largest cohort study with 22,013 patients using a nationwide database to compare OPD and MIPD, Robert et al. demonstrate that LPD is to be equivalent to OPD with regards to oncologic outcomes of margin status and lymph nodes removed [14]. This is in consistence with the conclusion of Adam et al. who used NCDB data and reported equivalent oncologic outcomes in patients undergoing OPD versus LPD for adenocarcinoma [33]. In the study of a large series of total LPD performed at the Mayo Clinic, Croome et al. compared the oncologic outcomes of 108 LPD with 214 OPD, performed for pancreatic adenocarcinoma, and found patients who underwent LPD had longer progression-free survival than patients who had OPD [21]. Given the previous reports that there is a decrease in survival with increasing time interval to adjuvant chemotherapy in patients with colon cancer and ovarian cancer [55, 56], and the fact that a significantly smaller proportion of patients received adjuvant chemotherapy after surgery until more than 8 weeks in the LPD group than OPD group, the authors suggested that the survival differences may be related to the ability to initiate or complete chemotherapy which is of great value to the oncologic outcome of the patients with malignant tumors. Also owing to the improved magnification and optics offered by the laparoscopic system, LPD allows surgeons to more precisely and accurately locate, control, and even dissect both small vessels and SMVs to prevent massive bleeding, thus leading to less blood loss during the operation and lowering transfusion rate. Considering that intraoperative blood transfusion is associated with tumor recurrence and metastasis, this may be another benefit to the oncologic outcomes of LPD [57]. Apart from those, it has also been shown in the reports that open surgery with huge traumatic stress suppresses the immune system by multiple mechanisms, and these effects appear to be much reduced with laparoscopy [58]. Considering the close relationship between immune dysfunction and tumor recurrence and metastasis [59], this may be also an oncologic advantage of LPD. Nevertheless, large prospective randomized controlled trials are still needed to further confirm the conclusion and the reasons remain to be substantiated.

In addition to the oncology results of LPD which we concern the most, there are some other benefits. For example, some authors believe that the less manipulation required by the laparoscopic surgery may be related to the reduced occurrence of postoperative adhesion and delayed gastric emptying (DGE). LPD can also have advantages for the postoperative combined use of enhanced recovery after surgery (ERAS) which was popular in recent years to facilitate faster recovery by virtue of lower rate of postoperative complications and minimally invasive. It has been reported that laparoscopic surgery within an ERAS protocol leads to better immunity preservation after surgery in colon carcinoma patients [60]. There are no similar reports on PD so far; large randomized controlled clinical trials about this should be performed in the future. And to eliminate the selection bias for the LPD group, the comparison between LPD an OPD should be performed only in patients who are suitable for the ERAS program.

The issue of cost is often considered to be an obstacle in the adoption of LPD because of the higher expenditure during the operation. However, Gerber et al. found the cost of LPD was equivalent to OPD in a study, and the total episode-of-care cost was even lower than OPD [61]. Tran et al. also showed that LPD was associated with a reduction in hospital costs in high-volume pancreatic centers in comparison with OPD [62]. This may be mainly due to a shorter length of hospital stay and less intensive ward costs. Therefore, though the current milieu of healthcare reform mandates better surgical performance and more accountability, the value of complex surgical procedures depends on outcomes achieved per dollar spent, and these studies show that cost should not be the problem for LPD.

Robotic surgery has gained tremendous attention because it has the potential to overcome many limitations of conventional laparoscopy, such as limited movement, unnatural positions for the surgeons, and so on [63]. However, despite some studies reported that the encouraging outcomes of robotic surgeries, comparisons between robotic and laparoscopic pancreatic surgery techniques and outcomes are lacking, and prospective randomized controlled trials are needed to further clearly define this issue. Considering that patients who have a conversion to laparotomy, no matter during LPD or RPD, have a poorer prognosis than totally LPD or RPD, it seems that this is an important merit of RPD compared with LPD according to some reports, but we should note this may mainly due to the high selective bias of the patients who underwent RPD considering the limited patients in the studies. The lack of tactile feedback seems to be a problem compared with laparoscopic surgery. Difficulties can arise during procedures with large operative fields such as PD, because they require changes of patients and instrument positions for retraction or exposure. The longer times for the robotic cases were anticipated given the additional time required to dock the robot, to exchange instruments, as well as to reposition or redock the instruments, if the viewing field necessitated change. A longer operation time means the prolonged anesthesia and pneumoperitoneum, thus leading to higher risks of cardiorespiratory failures during the operations. There are also authors who believe that the use of the robot even though it facilitates prompter learning of suturing skills, may actually limit the surgeon in training to achieve his/her potential by making him/her become dependent on the use of the robot for complex tasks [64].

In fact, as far as the author concerned, there is no question that the use of robots in surgery will continue to expand in the future, but long-term studies are still needed to demonstrate the overall equivalent oncologic outcomes, and patients who undergo the operation have to spend high expenditures, as well as the hospitals which introduce the equipment [44, 65]. In contrast, laparoscopic devices are much simpler and cheaper and are easier to popularize in most hospitals. Beyond that, as a machine, the robot always has the potential of malfunctions, though the rate is very low according to the reports [63]. And this will inevitably increase the insecurity of the operations to a certain extent. Therefore, there is no doubt that it will become an important tool in the surgical armamentarium in the future, but the extent of their use is still evolving. It is not easy to popularize among hospitals and patients at least in the coming future decades.

There were also reports that concluded different results about LPD. For example, in the report comparing practice patterns and short-term outcomes among 7061 patients, Adam et al. found that the 30-day mortality for patients undergoing MIPD was higher versus OPD, especially during the first phase of the learning curve [66]. In a recent report from Dutch, the clinical trial was even terminated by the data and safety monitoring board because of the more 90-day complication-related deaths associated with LPD [24]. However, as we know, hospital volume and learning curve play important roles in the outcomes of LPD. According to the report of Wang et al., the learning curve of LPD revealed three phases, with proficiency thresholds at 40 and 104 cases, respectively, and long learning curves, low-volume hospitals, and surgical inexperience were associated with higher rates of complications and mortality [67], and Boone et al. revealed similar results in their research [68], while in Adam’s study, the majority of hospitals performing MIPD were low volume centers, and in the clinical trial performed in Dutch, institutions, or surgeons that have done 20 or more LPDs can participate in the trial. Therefore, long learning curves, low-volume hospitals, and surgical inexperience may be associated with the higher rates of complications and mortality of LPD in the studies. In addition, two reports from the MD Anderson Cancer Center recently indicated that compared with open abdominal radical hysterectomy, the minimally invasive radical hysterectomy was associated with lower rates of disease-free survival and overall survival among women with early-stage cervical cancer [69, 70]. All these reports pose challenges to the perioperative and long-term survival outcomes of patients with minimally invasive operations for all malignant tumors including LPD. But we cannot deny previous researches about this issue and the use of minimally invasive techniques, including laparoscopic and robotic, in surgeries because of these reports. These reports should not be the obstacle, but let us become more cautious and rational while moving forward firmly. It should be noted that when we talk about a certain operation, it has not to be completely just one surgery solely. In some cases, in order to maximize the benefits of the surgery, we can combine the above two or more types of surgical methods, that is, the use of hand-assist ports or the hybrid technique. In addition, when finding it is too difficult to continue during the course of LPD or RPD, we should make a conversion to open surgery decisively. Therefore, we must pay attention to the actual situation and combine the different surgical methods reasonably, in order to optimize patient outcomes and mitigate unnecessary healthcare expenditures.

On the opposite, although the application of laparoscopic pancreatic surgery is increasing, we must also note that we should not blindly and complacently go for laparoscopic just for carrying out laparoscopy. In contrast, we should always remember that a competitive desire among surgeons and institutions to implement new minimally invasive techniques is to improve patients’ outcomes. Therefore, careful evaluation or assessment for each patient's situation comprehensively is indispensible. And the most suitable operation method should be chosen to maximize the patient's benefits.

For the surgeons, we should establish a better education system to minimize the steep learning curve of LPD. Access to education and training is still the key to popularize this minimally invasive surgical technique [71]. We believe that neither procedure is technically superior to the other, and whether having R0 resections with an adequate number of lymph nodes harvest largely depends on the technique and experience of the surgeon. Therefore, it is imperative to establish evidence-based guidelines with regard to the determination of competency and standardized training for surgeons with mandated supervision. Otherwise, the rapid introduction of LPD techniques could impair patient’s benefits and ultimately the success of the procedure. We should avoid similar mistakes when laparoscopic cholecystectomy was introduced, and some surgeons started performing the operation after only 1 to 3 days of training and without mandated supervision; as a result, there was a high likelihood of bile duct injury sustained among patients [72, 73]. In addition, according to a population-based analysis, risks of postoperative mortality and suboptimal oncologic surgical quality following PD are higher in low volume hospitals than in high volume centers and are more profound with LPD compared with OPD [74]. This reflects the importance of experience based on center volume to perform such complex operations as PD. Therefore, a more specialized and focused large pancreatic center may be more conducive to the development of pancreatic surgery and patients benefit.

Our department is one of the largest pancreatic tumor centers in China. A total of 40 cases of LPD and 50 cases of OPD had been implemented between September 2018 and March 2019; the pathologic characters of the patients include PDAC, ampullary tumor, IPMN, and other neoplasms in both groups. According to the preliminary data, the median length of hospital stay was 11 (9–34) days versus 12 (8–48) days and the median operation time was 383 (280–580) minutes versus 284 (230–470) min in the LPD and OPD group. While the median estimated blood loss was 100 (100–800) ml in the LPD group, which was significantly less than that in OPD group (260, 100–1200 ml) and previous reports, this may be related to the higher proportion of benign or borderline tumors, and the lesions are smaller (3.0, 1.5–7.5 cm) in our cohort. We will continue to expand our cohort and report the experience and outcome of our institution.

There are some limitations with this review which are mainly related to the quality of available data. The length of hospital stay is not only dependent on the recovery status and severity of postoperative complications, but also depends on the cultural background and medical insurance coverage of the patients in different institutions. Although in most cases, surgeons prefer performing LPD in patients with no comorbidity, no history of abdominal surgery, and no adiposity and benign or borderline neoplasms other than PDAC; almost all studies include all neoplasms mentioned above. There is no consensus on the criteria for patient selection so far. de Rooij et al. had raised a decision-aid algorithm for LPD, but it was based on their own experience and the limited available evidence [75]. Therefore, selection and publication bias unavoidably exists. Many of the studies included relatively a small number of patients, the data is from only a single center, and the follow-up is not very long. All these factors limit the generalizability of the results to some extent.

## Conclusions

To sum up, LPD is safe and feasible. The perioperative and long-term outcomes of LPD are no worse and even superior to OPD and RPD with regard to the length of hospital stay and estimated blood loss. Apart from strict control of indications, standardized training and learning should be guaranteed to ensure safe and stable development of LPD. Moreover, large prospective randomized controlled trials in multiple high volume centers are still needed to further confirm the outcomes. But we believe that the best times for it are coming.

## Abbreviations

IPMN: Intraductal papillary mucinous neoplasm
LPD: Laparoscopic pancreaticoduodenectomy
OPD: Open pancreaticoduodenectomy
PDAC: Pancreatic ductal adenocarcinoma
RPD: Robotic pancreaticoduodenectomy
